# Whole-exome sequencing of rectal cancer identifies locally recurrent mutations in the Wnt pathway

**DOI:** 10.18632/aging.203618

**Published:** 2021-10-12

**Authors:** Yi Yang, Xiaodong Gu, Zhenyang Li, Chuang Zheng, Zihao Wang, Minwei Zhou, Zongyou Chen, Mengzhen Li, Dongbing Li, Jianbin Xiang

**Affiliations:** 1 Department of General Surgery, Huashan Hospital, Fudan University, Shanghai 200040, China; 2 MyGene Diagnostics Co., Ltd, Guangzhou 510000, China

**Keywords:** locally recurrent rectal cancer, whole-exome sequencing, genetic variation, Wnt signaling pathway, precision medicine

## Abstract

Locally recurrent rectal cancer (LRRC) leads to a poor prognosis and appears as a clinically predominant pattern of failure. In this research, whole-exome sequencing (WES) was performed on 21 samples from 8 patients to search for the molecular mechanisms of LRRC. The data was analyzed by bioinformatics. Gene Expression Profiling Interactive Analysis (GEPIA) and Human Protein Atlas (HPA) were performed to validate the candidate genes. Immunohistochemistry was used to detect the protein expression of LEF1 and CyclinD1 in LRRC, primary rectal cancer (PRC), and non-recurrent rectal cancer (NRRC) specimens. The results showed that LRRC, PRC, and NRRC had 668, 794, and 190 specific genes, respectively.

*FGFR1* and *MYC* have copy number variants (CNVs) in PRC and LRRC, respectively. LRRC specific genes were mainly enriched in positive regulation of transcription from RNA polymerase II promoter, plasma membrane, and ATP binding. The specific signaling pathways of LRRC were Wnt signaling pathway, gap junction, and glucagon signaling pathway, etc. The transcriptional and translational expression levels of genes including NFATC1, PRICKLE1, SOX17, and WNT6 related to Wnt signaling pathway were higher in rectal cancer (READ) tissues than normal rectal tissues. The *PRICKLE1* mutation (c.C875T) and *WNT6* mutation (c.G629A) were predicted as “D (deleterious)”. Expression levels of LEF1 and cytokinin D1 proteins: LRRC > PRC > NRRC > normal rectal tissue. Gene variants in the Wnt signaling pathway may be critical for the development of LRRC. The present study may provide a basis for the prediction of LRRC and the development of new therapeutic drugs.

## INTRODUCTION

Locally recurrent rectal cancer (LRRC) is defined as the recurrence of rectal cancer (READ) only within the pelvis after radical resection [1]. LRRC is associated with symptoms of significant morbidity, such as rectal bleeding, bowel obstruction, chronic pain, fistulas, malodorous tumor discharge, tenesmus, and pelvic sepsis [2, 3]. LRRC generally occurs within 2-3 years after the initial surgery [4]. Non-recurrent rectal cancer (NRRC) is defined as READ that has not recurred within 3 years after radical surgery. Historically, the introduction of total mesenteric excision (TME) has greatly reduced the local recurrence rate from 30% to 10% [3]. Combined with chemoradiotherapy, the local control (LC) rate for clinically localized READ ranges between 92% and 96% [5, 6]. Up to 50% of recurrent READ patients have synchronously diagnosed distant metastases [7, 8]. Meanwhile, nearly half of the patients with recurrence of READ are limited in the pelvis, which is defined as LRRC [8–10].

The causes and mechanism of LRRC have become a focus of current clinical medical research, highlighting the need for prevention of LRRC. Risk factors for LRRC occurrence include anastomotic leak, incomplete resection, intraoperative tumor perforation, high-grade pathology, and lack of adjuvant chemoradiotherapy [11]. LRRC is associated with short-term mortality, high reoperation rate, and additive healthcare costs [12]. Without treatment, the life expectancy of LRRC patients is limited and the quality of life is usually poor. Three major strategies for reducing the recurrence of READ are wider surgical coverage and neoadjuvant radiotherapy and neoadjuvant chemotherapy [13, 14]. However, neoadjuvant chemoradiation may significantly increase morbidity. Since LRRC portends a significantly worse oncologic outcome and quality of life, remarkable advances in surgical techniques and adjuvant chemoradiotherapy are improving the overall survival (OS) of LRRC. Although modern surgical techniques and multimodal therapies have proved to be effective, LRRC shows a significant treatment challenge. Some research showed a 5-year survival rate of 35% to 50% after surgery for LRRC [15, 16]. Chemotherapy alone can prolong OS to12 to 15 months, but it is not curable [8]. About 40% of LRRC patients have an opportunity for surgical treatment, but performing the operation can be a challenging option under such a situation [17]. Reoperation is very difficult and often needs to be combined with organ resection. The incidence of perioperative complications is specifically high, which is rare in domestic medical units. Therefore, how to prevent LRRC is the key to improve the survival and life quality of READ patients.

The long-term outcome of surgical treatment mainly depends on the clear margin resection [16]. We speculated that the molecular mechanism of LRRC may also be related to the occurrence and prognosis of LRRC. The signal pathway related to distant metastasis of READ has been studied in detail as a hot spot. However, the molecular mechanism of LRRC has not been reported yet. Whole-exome sequencing (WES) can reveal mutations within exon coding regions. This study was based on WES to explore mutations associated with LRRC. 5 trios of LRRC and 3 pairs of NRRC samples were sequenced by WES. The data was analyzed systematically by bioinformatics. Gene Expression Profiling Interactive Analysis (GEPIA) and Human Protein Atlas (HPA) were performed to validate the candidate genes. Immunohistochemistry was used to detect the protein expression of candidate genes in LRRC, PRC, and NRRC specimens. Our findings provided a basis for predicting LRRC and developing new therapeutic agents.

## MATERIALS AND METHODS

### Patients and specimens

The present study was reviewed and approved by the Ethics Committee of Huashan Hospital affiliated to Fudan University. We collected 21 samples, including 15 tissue samples from five LRRC patients (PRC, adjacent normal rectal tissue, and LRRC) and three NRRC patients (NRRC and adjacent normal rectal tissue) from May 2019 to November 2019 at Huashan Hospital, Fudan University. HE-stained sections from each sample were subjected to an independent pathology review to confirm that the tumor specimens were histologically consistent with LRRC and that the adjacent tissue specimens contained no tumor cells. All participants provided written informed consent for genetic analysis.

### Whole-exome sequencing

For every individual, the genomic DNA of cells from one or two regions of the PRC, LRRC, and matched normal rectal tissue samples were sequenced. DNA was extracted from formalin-fixed and paraffin-embedded (FFPE) tissue blocks using MagPure FFPE DNA/RNA LQ Kit (Magen, Guangzhou, China). The DNA was then subjected to an additional quality and quantity evaluation step by utilizing NanoDrop ultra-micro spectrophotometer (Thermo Fisher Scientific, Wilmington, DE, USA) and Qubit 1X dsDNA HS Assay Kit (Thermo Fisher Scientific, Wilmington, DE, USA). Exome-coding DNA was captured with Agilent SSEL XT HS Human All Exon V6 (Agilent Technologies, Santa Clara, CA, USA), and the libraries were sequenced on an Illumina NovaSeq Platform (Illumina, San Diego, CA, USA), which produced 350-bp paired-end reads.

### Sequencing data analysis

Raw sequencing data were filtered using the Trim Galore program to remove low quality reads at both ends of the sequencing [18]. The FastQC package (http://www.bioinformatics.babraham.ac.uk/projects/fastqc) was used to assess the quality score distribution of the sequencing reads. Read sequences were mapped to the human reference genome (GRCH37/hg19) using Burrows-Wheeler Aligner (BWA, v.0.7.15) with the default parameters, and duplicates were marked and discarded using Picard (http://broadinstitute.github.io/picard). After alignment by BWA, the reads were subjected to local realignment and recalibration using the Genome Analysis Toolkit (GATK). Variants and genotypes calling were also performed using Genome Analysis Toolkit (GATK, v3.7).

Point substitutions in all tumor samples were counted and the proportion of each point substitution (C> A, C> G, C> T, T> A, T> C, and T> G) was calculated for both PRC and LRRC samples. The results were visualized by GraphPad Prism 8.

ANNOVAR was utilized to annotate all called variants. The SNVs of the adjacent normal rectal tissue in PRC, LRRC, and NRRC samples were removed for further analysis. Mutations were screened before being subjected to pathway enrichment analysis. The minor allele frequency (MAF) of variants was evaluated in the 1000 Genome Project (http://www.ncbi.nlm.nih.gov/variation/tools/1000genomes/), ExAC (http://exac.broadinstitute.org/), variants of MAF<1% and annotated “.” (No annotation information in the database) were retained. Subsequently, the pathogenicity of variants was predicted according to SIFT (http://sift.jcvi.org), Polyphen2 (http://genetics.bwh.harvard.edu/pph2) and FATHMM (http://fathmm.biocompute.org.uk/). Variants predicted by the three tools as “D (damaging)” were retained.

CNVs were identified using open source software called CNVkit (v0.9.7), a tool kit to infer and visualize the copy numbers from targeted DNA sequencing data. Genes of copy number over “3” were selected, the copy number of “2” meant no CNV, the copy number over “2” meant a copy gain in some paired chromosomes. The CNVs of the adjacent normal rectal tissue in PRC, LRRC, and NRRC samples were removed for further analysis.

### Enrichment analyses of gene ontology (GO) and the Kyoto Encyclopedia of Genes and Genomes (KEGG)

The specific genes of PRC, LRRC, and NRRC samples were used to perform GO and KEGG enrichment analysis by DAVID 6.8 (https://david.ncifcrf.gov/). The results were considered as statistically significant if *P* value< 0.05*.* The top 15 enriched GO terms were visualized by BMKCloud (http://www.biocloud.net/), a free online platform for data analysis. The top 15 KEGG pathways were visualized by R ggplot2 package. GO functional analysis was divided into three parts: biological process (BP), cellular component (CC), and molecular function (MF).

### Mutations of driver genes

To look for potential LRRC drivers, mutations in 155 TCGA rectal adenocarcinoma (https://www.cbioportal.org/) and 339 MSK READ patients were included in the analysis for comparison. Forty-eight genes with a high frequency of occurrence in both cases (TCGA Freq ≥ 10%; MSK Freq ≥ 5%) were selected. The mutation patterns of these genes in PRC and LRRC tissues were then compared.

### mRNA and protein expression levels of hub genes

To validate the expression of the key DEGs, the Gene Expression Profiling Interactive Analysis (GEPIA) website (http://gepia2.cancer-pku.cn/#index) was applied to analyze the data of RNA sequencing expression based on thousands of samples from the GTEx project and TCGA [19]. The association between overall survival (OS) and the genes expressed in GC patients was determined using GEPIA. The lower and upper 50% of gene expression were set as the standard for analysis. Log-rank test results with P<0.05 were regarded as statistically significant. Besides, the GEPIA was employed to visualize the mRNA expression of hub genes in tumors and normal samples.

The Human Protein atlas (HPA) database (https://www.proteinatlas.org/) is a free online database that provides abundant transcriptome and proteome data on human normal or pathological tissues through RNA-sequence analysis and immunohistochemical analysis. In the present study, the protein expression and distribution of hub genes were investigated in GC tissues and compared with normal tissues in HPA [18].

### Immunohistochemistry

The Formalin-Fixed and Paraffin-Embedded (FFPE) samples were used in this study. 3-mm tumor sections were incubated with commercial rabbit polyclonal antibodies against LEF1 (ab137872, abcam) and cyclin D1 (ab16663, abcam) at 1/100 dilution overnight at 4° C. Then, the sections were conjugated with HRP-Sheep Anti-Rabbit IgG-HRP-Sheep Anti-Mouse IgG antibody (BOSTER BA1056, 1:500 dilution;) at room temperature for 2 h, then covered by 3, 3-diaminobenzidine (DAB) (Vector Laboratories, Burlingame, CA). Elivision plus kit for immunohistochemistry (IHC) (KIT-990, MXB) was used in this study.

### Statistical analyses

Fisher’s exact test was used to assess differences in the count data. Top 15 significant GO terms of BP, MF, and CC were listed according to the p-value. Top 15 significant KEGG pathways were listed according to the p-value. P<0.05 was considered statistically significant.

## RESULTS

### WES data

The clinical characteristics of the eight patients are listed in Supplementary Table 1. Age range is 45-71 years. The gender included 8 males. The tumor size included 4 patients (2cm), 3 patients (3cm), and 1 patient (4cm), and 1 patient (5cm). The pathologic 4 stage I, 3 stage II, and 1 stage III. The sequencing quality of the WES was analyzed and the raw data, Q30 (proportion of mapped reads) and mean depth of each sample are shown. As shown in Supplementary Table 2, all samples had Q30 ratio >90%, good sequencing quality, >90% of mapped reads, and an average sequencing depth > 200 x, which was sufficient to identify mutations.

### Mutational signatures of LRRC and PRC tumors

**Figure 1:**
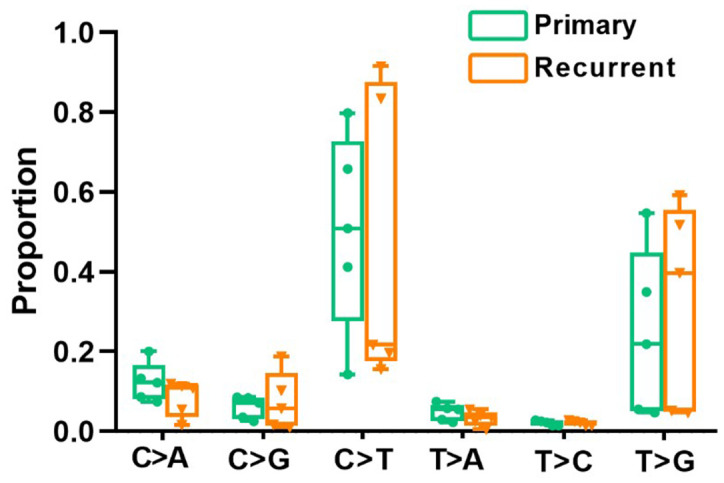
**Divergent mutational features in LRRC and PRC samples of LRRC patients.** Six mutational subtypes in LRRC and PRC tissues. LRRC tissues are presented in orange, and the PRC tissues are presented in cyan.

All point substitutions were divided into six groups (C>A, C>G, C>T, T>A, T>C and T>G) according to the direction of the mutation. In total, 97862 and 47292 somatic substitutions were identified in all PRC and LRRC tissues by comparing them with the matched adjacent normal samples. The proportion of each group was different, but the proportion of all six mutant groups did not differ significantly between LRRC and matched PRC tissues (Figure 1).

### The common variations in LRRC and PRC tumors

After removing the control SNVs from PRC and LRRC samples, the focus was on common variations. In PRC, four genes, including *MYCN, SCRIB, SNAPC4, and MED15*, were identified with eight mutations (Table 1). Notably, the SCRIB mutation (c.T233G) was predicted as “D (deleterious)” by SIFT and Polyphen2 software. In the LRRC, two genes, including *SCRIB* and *RUSC2* (Table 2), were found to have three mutations. Interestingly, LRRC had the same *SCRIB* mutation as PRC. In addition, two frame shift mutations were found in the *RUSC2* gene (c.831_833del, c.3465_3467del).

**Table 1:** Common variations in PRC samples.

**Gene**	**Mutation type**	**Variants**	**1000g2015_eas**	**ExAC eas**	**SIFT**	**Polyphen2**
MYCN	Frame shift insertion	c.87dupC	-	-	-	-
SCRIB	Nonsynonymous	c.233T>G	-	-	D	D
SNAPC4	Nonsynonymous	c.3158T>A	-	-	D	B
MED15	Synonymous	c.1035G>C	-	-	-	-
		c.1197G>C	-	-	-	-
		c.1170G>C				
		c.1248G>C				
		c.1368G>C				

**Table 2:** Common variations in LRRC samples.

**Gene**	**Mutation type**	**Variants**	**1000g2015_eas**	**ExAC eas**	**SIFT**	**Polyphen2**
SCRIB	Nonsynonymous	c.233T>G	-	-	D	D
RUSC2	Non-frameshift deletion	c.831_833del c.3465_3467del	-	-	-	-

### The biological process and pathways related to PRC, LRRC, and NRRC

**Figure 2:**
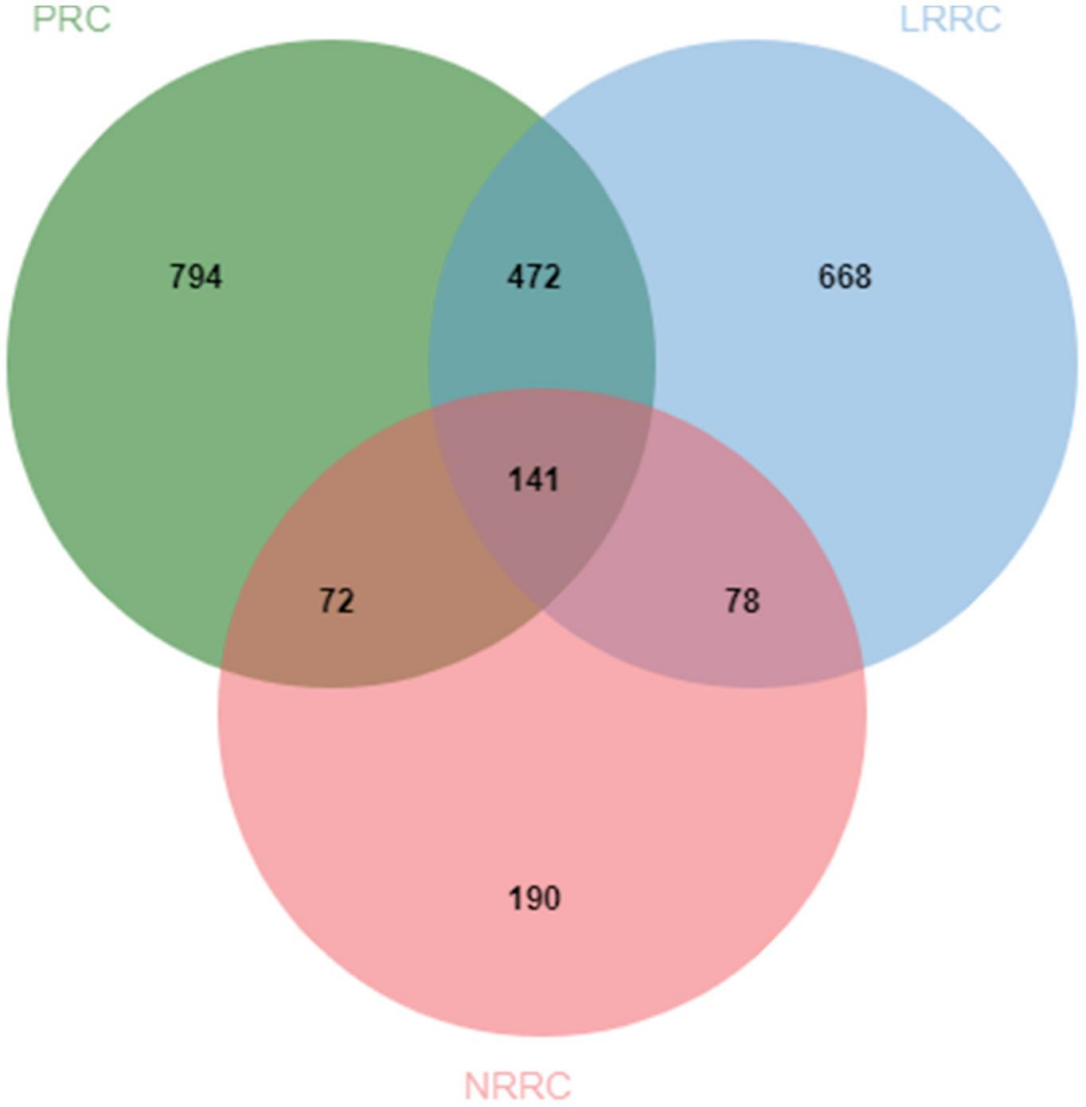
**Venn diagram of likely pathogenic genes associated with PRC, LRRC, and NRRC.** PRC, primary rectal cancer; NRRC, non-recurrent rectal cancer; LRRC, local recurrent rectal cancer.

In the PRC samples, there were 110,198 variants after removing the control mutations, 2388 variants after screening, and 1479 genes after further deduplication. In the LRRC samples, there were 100061 variants after removing the control variants, 2076 variants after screening, and 1360 genes after further deduplication. In the NRRC samples, there were 23067 variants after removing the control variants, 573 variants after screening, and 481 genes after further deduplication. The PRC, LRRC, and NRRC had 794, 668, and 190 specific genes, respectively (Figure 2). There were 472 likely pathogenic genes shared by the PRC and LRRC samples. The genes including 668 genes and 472 genes were important for the development of LRRC.

**Figure 3:**
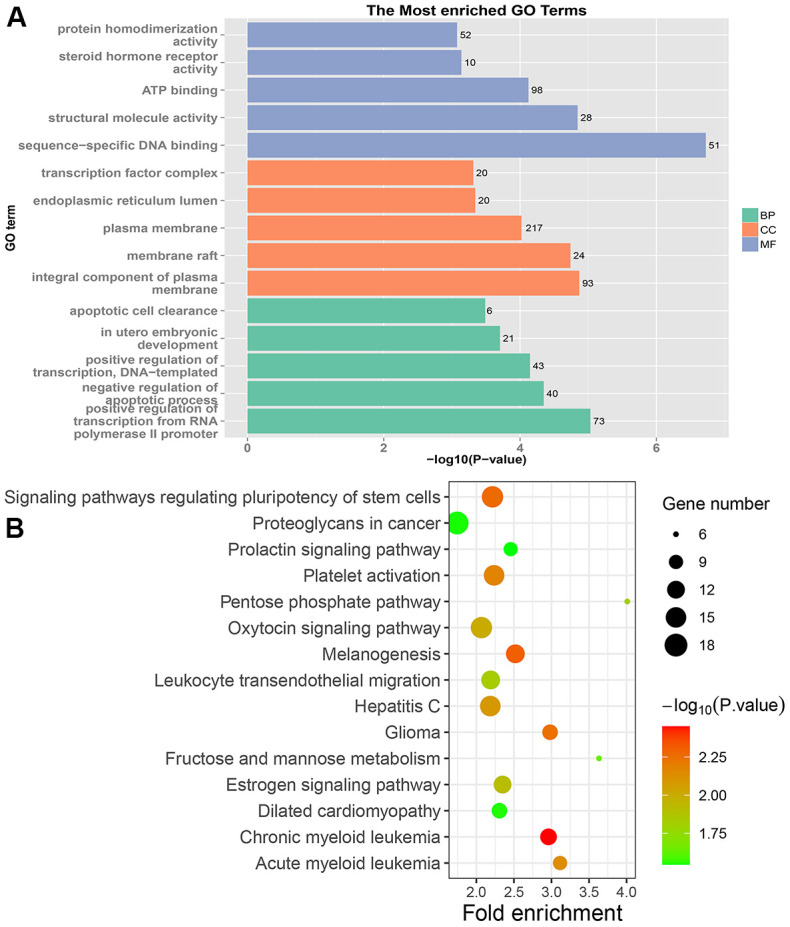
**GO and KEGG enrichment analysis of PRC samples.** (**A**) The top 15 enriched GO terms. (**B**) The top 15 significant KEGG pathways. Fold enrichment represents the degree of enrichment. The size of the bubble indicates the number of genes. The depth of bubble color indicates the level of significance.

**Figure 4:**
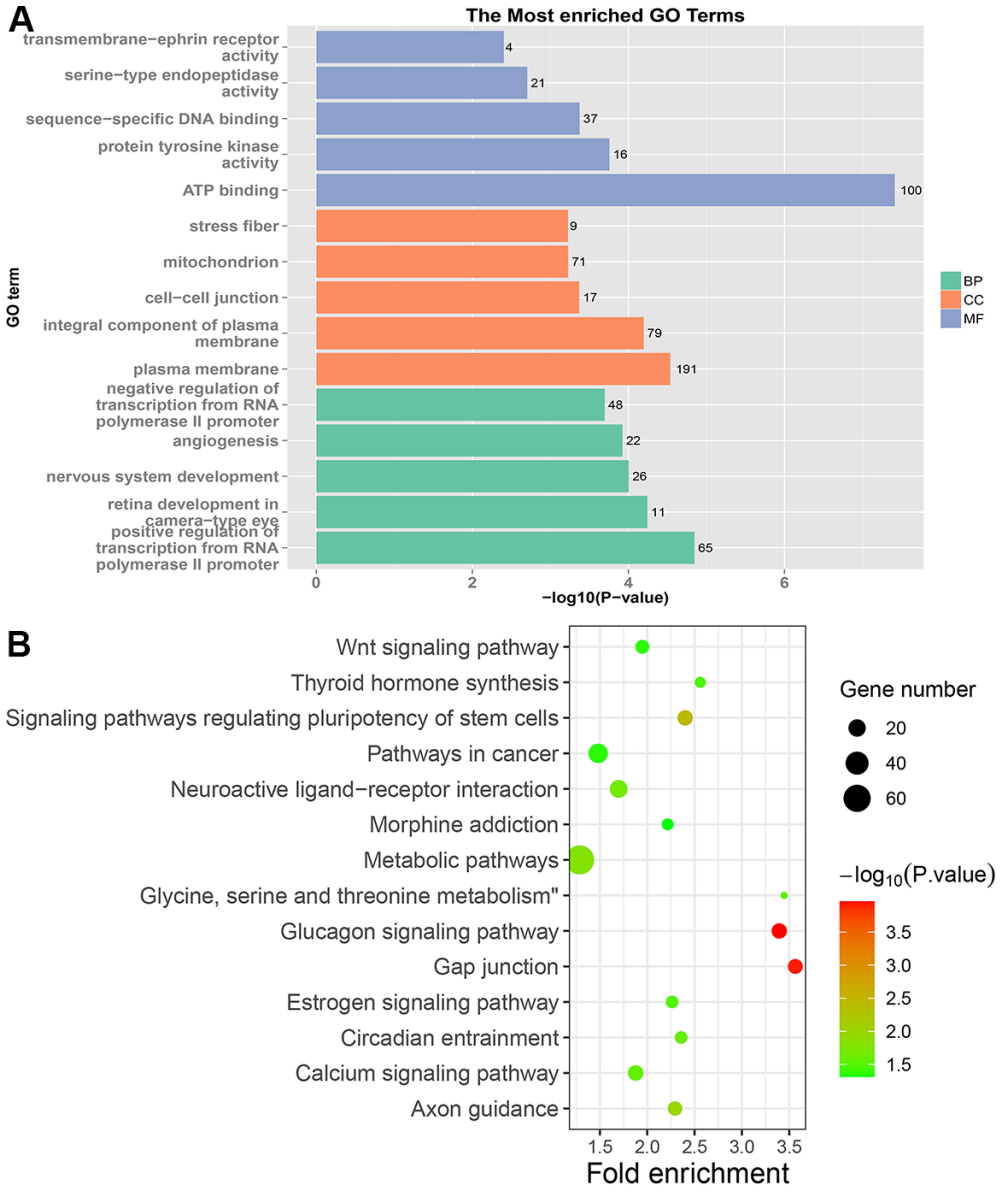
**GO and KEGG enrichment analysis of LRRC samples.** (**A**) The top 15 enriched GO terms. (**B**) The top 15 significant KEGG pathways. Fold enrichment represents the degree of enrichment. The size of the bubble indicates the number of genes. The depth of bubble color indicates the level of significance.

**Figure 5:**
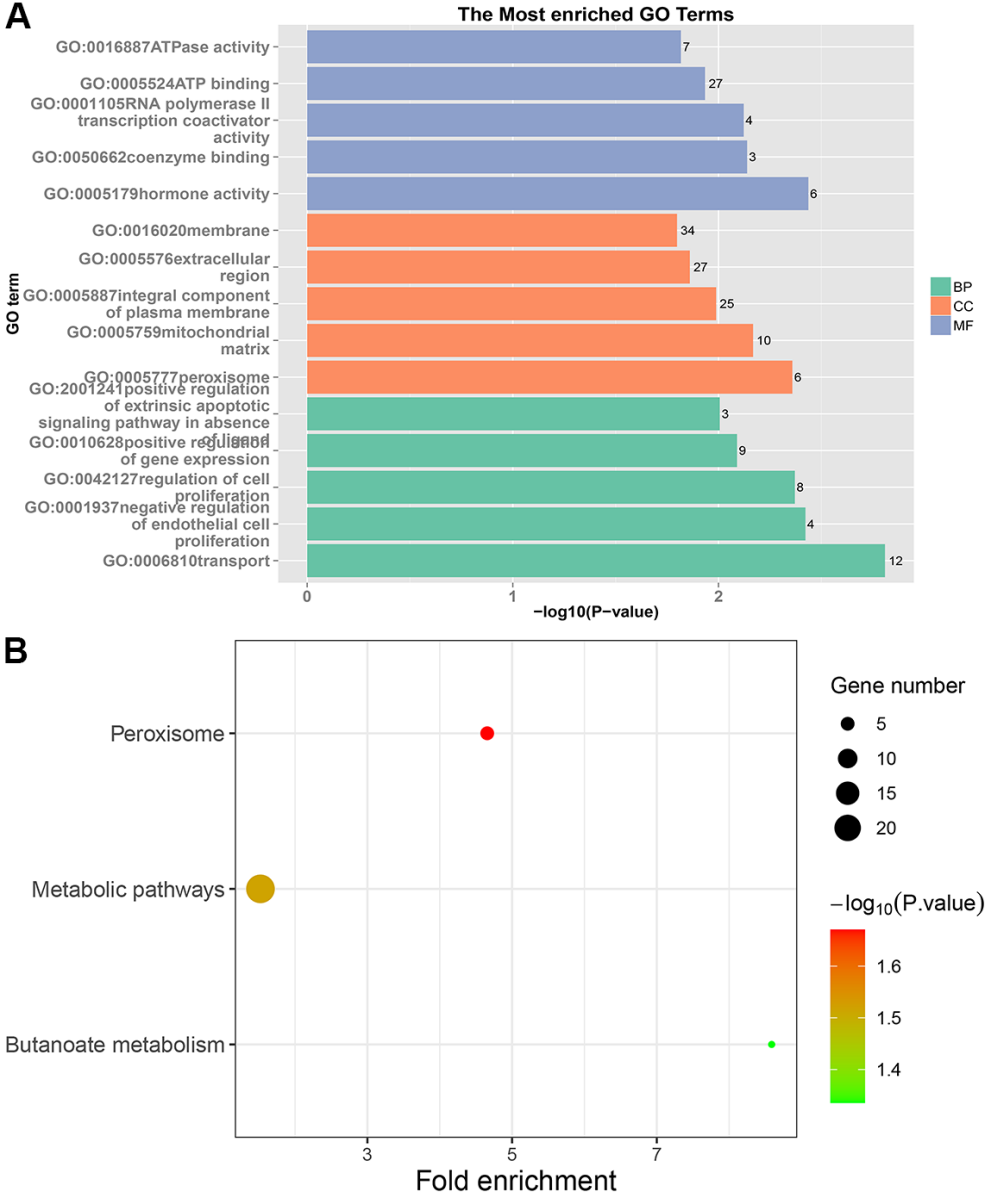
**GO and KEGG enrichment analysis of NRRC samples.** (**A**) The top 15 enriched GO terms. (**B**) The top 15 significant KEGG pathways. Fold enrichment represents the degree of enrichment. The size of the bubble indicates the number of genes. The depth of bubble color indicates the level of significance.

In PRC samples, GO functional analysis showed that the specific genes were mainly enriched in the biological process of positive regulation of negative regulation of apoptotic process and apoptotic cell clearance (Figure 3A and Supplementary Table 3). The genes were mainly enriched in signaling pathways such as proteoglycans in cancer, ECM-receptor interaction, ErbB signaling pathway, and protein digestion and absorption (Figure 3B and Supplementary Table 4). In LRRC samples, specific genes were mainly enriched in the biological process including angiogenesis and regulation of transcription from RNA polymerase II promoter (Figure 4A and Supplementary Table 5). The genes were mainly enriched in the signaling pathways such as glucagon signaling pathway, gap junction, axon guidance, and metabolic pathways (Figure 4B and Supplementary Table 6). In NRRC samples, specific genes were mainly enriched in the biological process including regulation of cell proliferation and positive regulation of extrinsic apoptotic signaling pathway in the absence of ligand (Figure 5A and Supplementary Table 7). The genes were mainly enriched in the signaling pathways such as peroxisome, metabolic pathways, and butanoate metabolism (Figure 5B and Supplementary Table 8).

### Wnt signaling pathway is one of the specific pathways in LRRC

**Figure 6:**
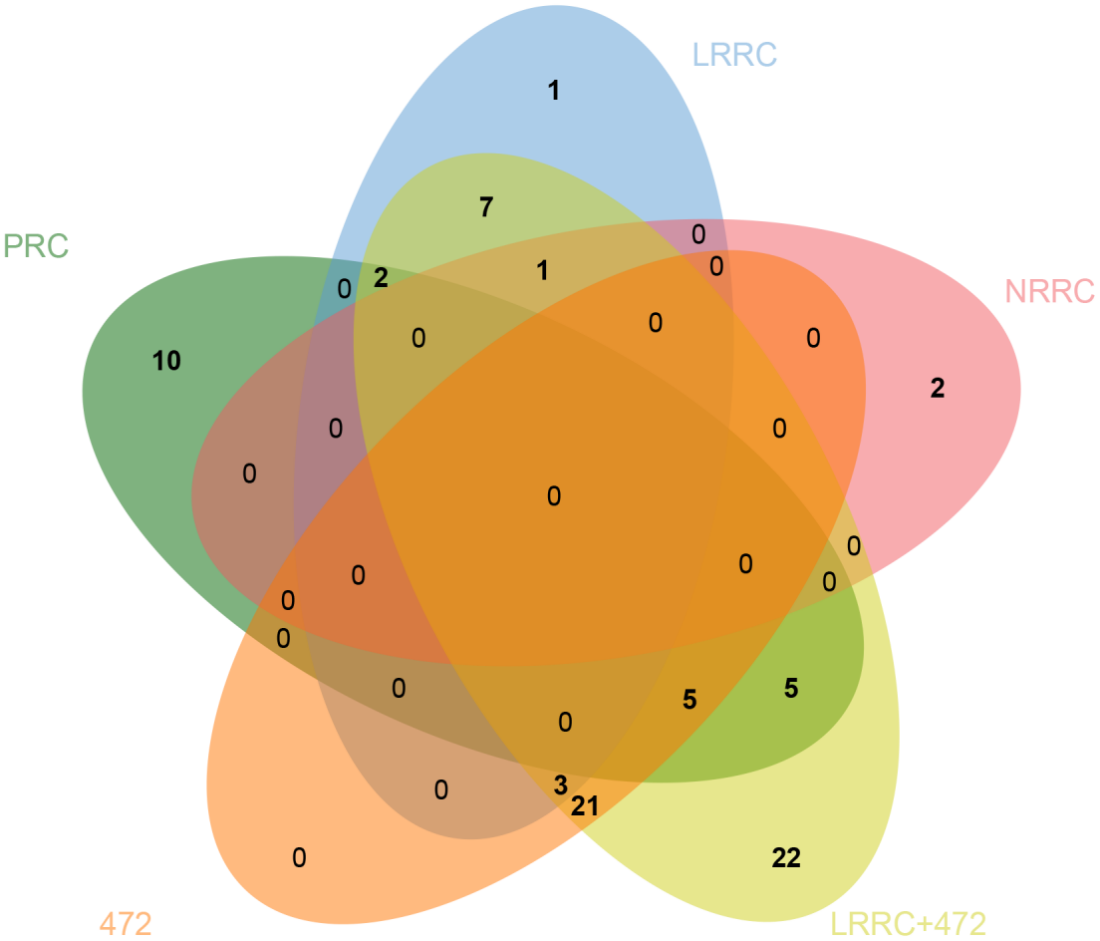
**Venn diagram of specific pathways in PRC, LRRC, NRRC, 472, and LRRC+472.** 472, 472 common genes; LRRC+472, LRRC+ 472 common genes.

Venn analysis of the enriched KEGG pathway was performed on five sets of samples. As shown in Figure 6, there are 7 specific pathways in LRRC, including Wnt signaling pathway, Gap junction, Glucagon signaling pathway, Axon guidance, Thyroid hormone synthesis, Morphine addiction and Glycine, serine and threonine metabolism.

### The variations of genes related to Wnt signaling pathway in LRRC

In the present study, 24 genes were enriched in Wnt signaling pathway of LRRC samples (Table 3). And 12 genes were enriched only in Wnt signaling pathway of LRRC samples (Supplementary Table 6). The variations of the 12 genes were listed in Table 4. 15 variations of the 12 genes were predicted as “D (deleterious)” by SIFT, Polyphen2, and FATHMM software, which were critical to the development of LRRC.

**Table 3:** The specific pathways in LRRC samples.

**Term**	**Count**	**%**	**P-Value**
hsa04310: Wnt signaling pathway	24	0.013840352	0.000623
hsa04540: Gap junction	23	0.01326367	0.000001
hsa04922: Glucagon signaling pathway	22	0.012686989	0.000032
hsa04360: Axon guidance	22	0.012686989	0.001176
hsa04918: Thyroid hormone synthesis	16	0.009226901	0.000375
hsa05032: Morphine addiction	15	0.00865022	0.013851
hsa00260: Glycine, serine and threonine metabolism	9	0.005190132	0.011322

**Table 4:** Variations of genes related to Wnt signaling pathway in LRRC Samples.

**Gene**	**Mutation type**	**AAChange**	**SIFT**	**FATHMM**	**Frequency**
CHD8	nonsynonymous	NM_001170629: exon30: c.G5564T: p.R1855L	D	D	0.012788
CHD8	nonsynonymous	NM_001170629: exon17: c.G3542A: p.G1181E	D	D	0.051282
FZD5	nonsynonymous	NM_003468: exon2: c.C728T: p.S243L	D	D	0.019231
FZD5	nonsynonymous	NM_003468: exon2: c.C155T: p.P52L	D	D	0.042857
NFATC1	nonsynonymous	NM_001278673: exon4: c.C236T: p.T79M	D	D	0.015842
PLCB3	nonsynonymous	NM_001184883: exon15: c.G1736A: p.R579H	D	D	0.018927
PRICKLE1	nonsynonymous	NM_001144881: exon7: c.C875T: p.P292L	D	D	0.051471
PRICKLE2	nonsynonymous	NM_198859: exon7: c.A908C: p.Q303P	D	D	0.023973
PRICKLE2	nonsynonymous	NM_198859: exon8: c.G2081A: p.R694H	D	D	0.010539
PRKACA	nonsynonymous	NM_001304349: exon6: c.G727A: p.D243N	D	D	0.036364
SMAD4	nonsynonymous	NM_005359: exon11: c.C1373T: p.A458V	D	D	0.035088
SOX17	nonsynonymous	NM_022454: exon2: c.G1075A: p.D359N	D	D	0.034483
TCF7L1	nonsynonymous	NM_031283: exon8: c.A920C: p.H307P	D	D	0.05291
VANGL2	nonsynonymous	NM_020335: exon2: c.C23T: p.S8L	D	D	0.023729
WNT6	nonsynonymous	NM_006522: exon3: c.G629A: p.G210D	D	D	0.055046

### Analysis of the genes related to Wnt signaling pathway via GEPIA and HPA databases

**Figure 7:**
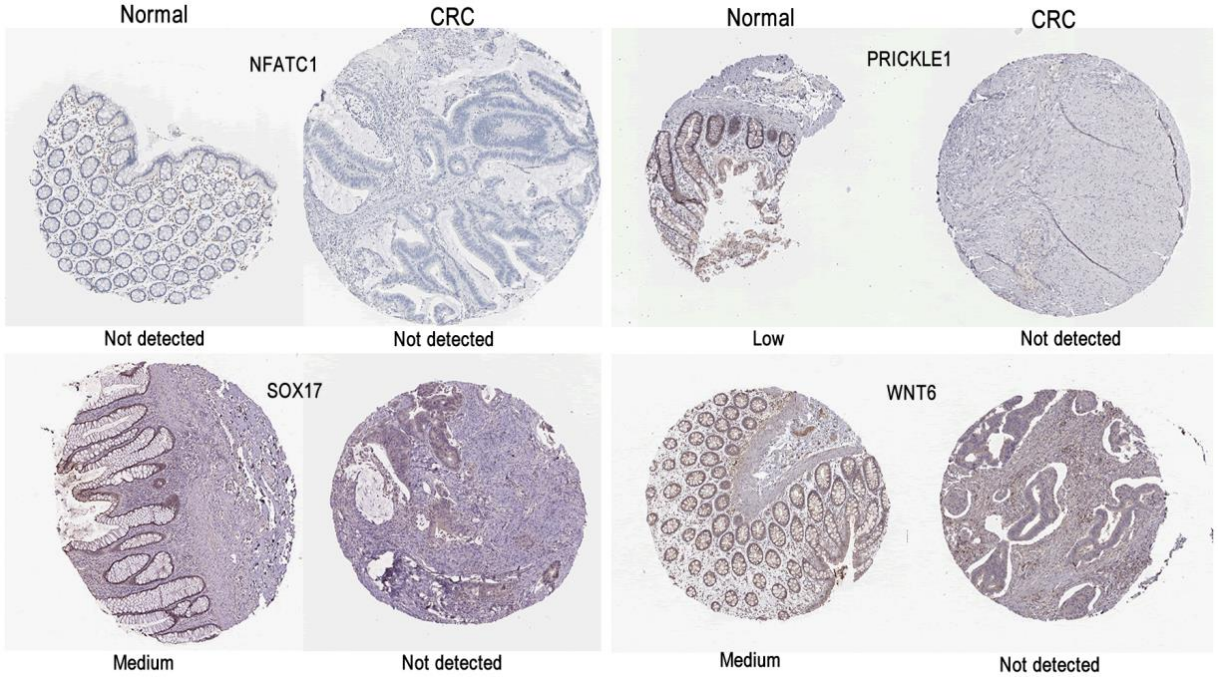
**Validation of the mRNA expression levels of *NFATC1, PRICKLE1, SOX17,* and *WNT6* in READ tissues and normal rectal tissues using GEPIA.** The red box represents READ samples (92), and the gray box represents normal samples (318). READ, rectal adenocarcinoma. Significance markers: ***, p<0.001.

**Figure 8:**
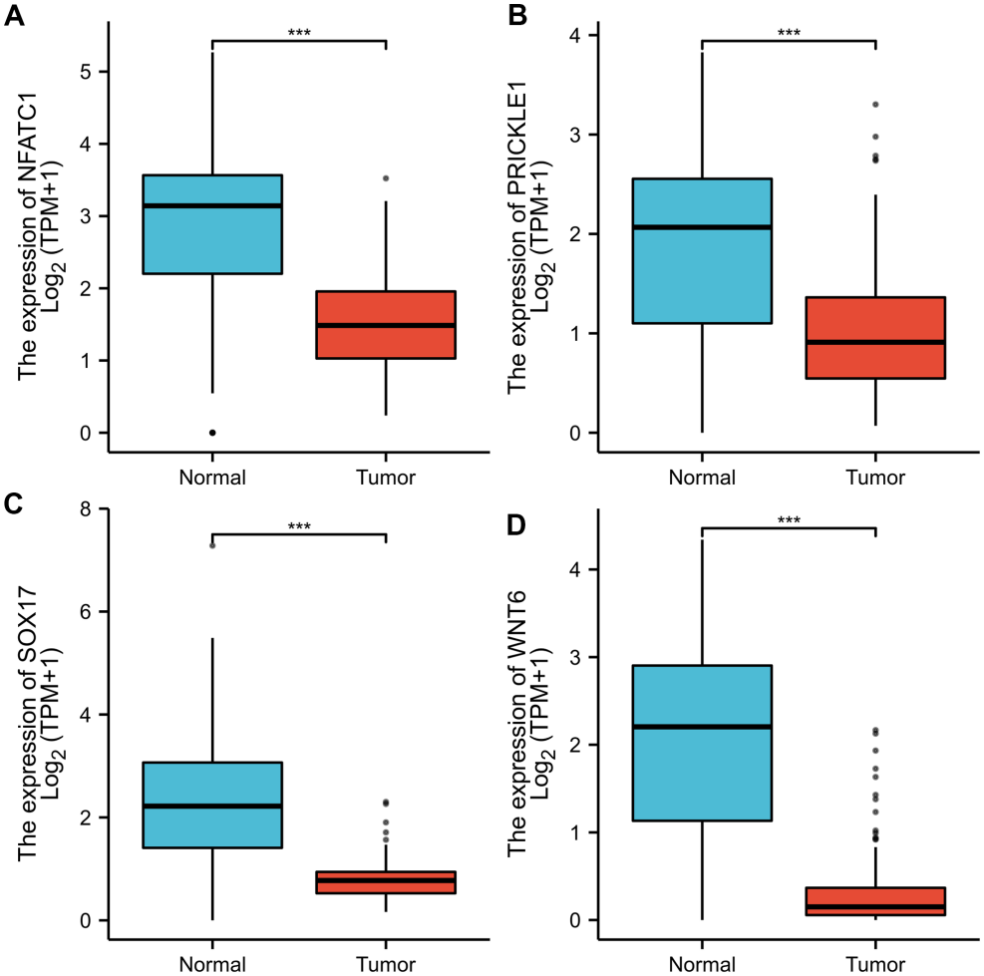
Representative immunohistochemistry images of (**A**) NFATC1; (**B**) PRICKLE1; (**C**) SOX7; (**D**) WNT6 in CRC and colorectal tissues derived from the HPA database. HPA, Human Protein Atlas.

**Figure 9:**
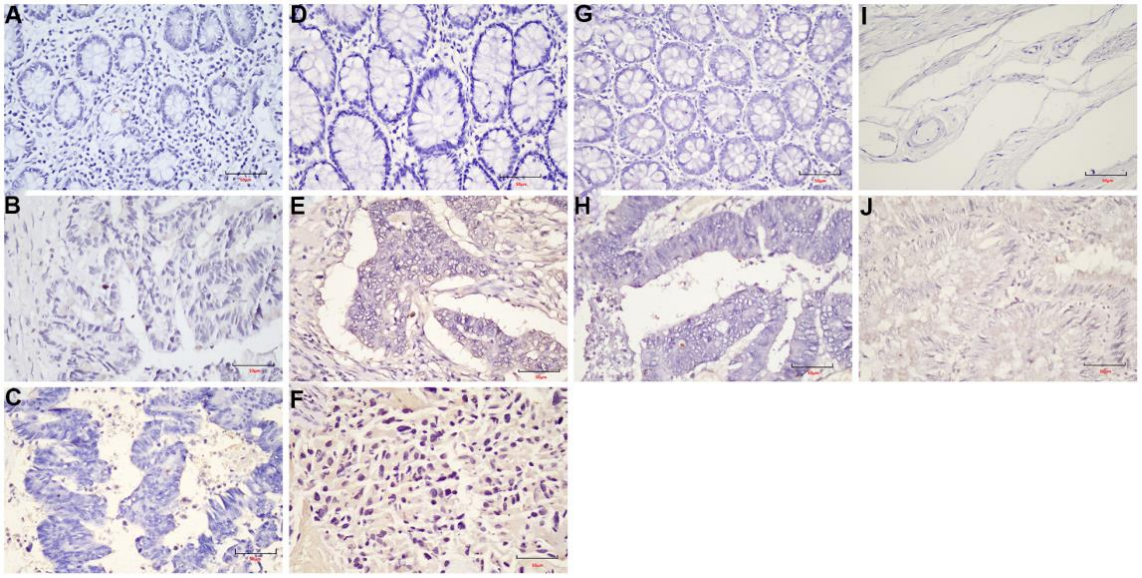
**The expression of *LEF1* in FFPE samples.** (**A**) DST-LRRC; (**B**) DST-PRC; (**C**) DST-rectal tissue; (**D**) LZQ-LRRC; (**E**) LZQ-PRC; (**F**) LZQ-rectal tissue; (**G**) LHC-NRRC; (**H**) LHC-rectal tissue; (**I**) SCC-NRRC; (**J**) SCC-rectal tissue. Scale bar=50μm.

**Figure 10:**
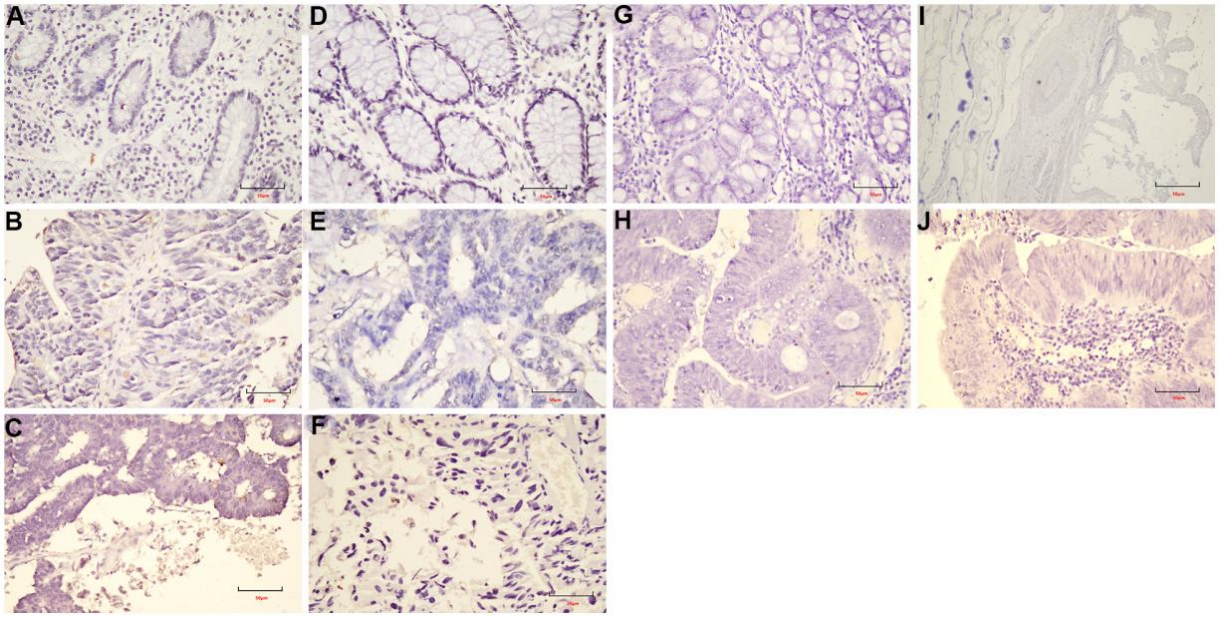
**The expression of *cyclin D1* in FFPE samples.** (**A**) DST-LRRC; (**B**) DST-PRC; (**C**) DST-rectal tissue; (**D**) LZQ-LRRC; (**E**) LZQ-PRC; (**F**) LZQ-rectal tissue; (**G**) LHC-NRRC; (**H**) LHC-rectal tissue; (**I**) SCC-NRRC; (**J**) SCC-rectal tissue. Scale bar=50μm.

As shown in Figure 7, the expression levels of *NFATC1, PRICKLE1, SOX17,* and *WNT6* in CRC tissues were significantly lower than in normal tissues. The protein expression levels of the genes in CRC were explored using the HPA database (Figure 8). The protein levels of *NFATC1* were not expressed in CRC tissues and normal tissues. The protein level of PRICKLE1 was not expressed in CRC tissues, whereas the low protein expression level of PRICKLE1 was observed in normal tissues. The protein levels of SOX17 and WNT6 were not expressed in CRC tissues, whereas the low protein expression levels of SOX17 and WNT6 were observed in normal tissues. In summary, the present results indicated that the transcriptional and translational expression levels of the hub genes were overexpressed in patients with CRC. *LEF1* and *cyclin D1* are key genes in the Wnt signaling pathway. IHC was performed to test LEF1 and cyclin D1 protein expression in adjacent normal rectal tissue, PRC and LRRC FFPE samples. As shown in Figures 9, 10, the expression of LEF1 and cyclin D1 proteins was higher in PRC compared to adjacent normal rectal tissue; the expression of LEF1 and cyclin D1 proteins was higher in LRRC compared to PRC; and the expression of LEF1 and cyclin D1 proteins was higher in PRC compared to NRRC. The above results suggest that the Wnt signaling pathway may play an important role in the development of LRRC.

### Analysis of driver genes in LRRC

**Figure 11:**
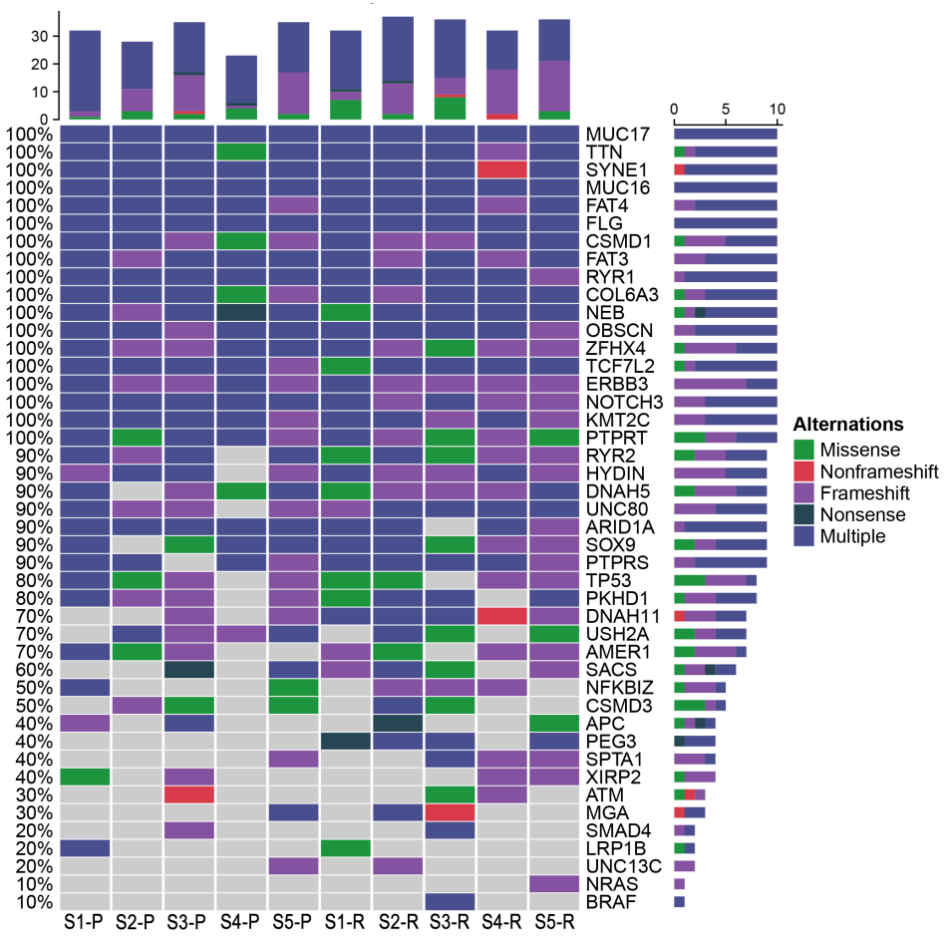
**Potential driver genes related to LRRC.** Forty-eight genes included (1) genes reported as significantly mutated genes in the previous study in CRC; (2) genes with a higher mutation rate in the cohort (TCGA Freq≥10%; MSK Freq≥5%).

Based on the database, 48 possible driver genes were selected. As shown in Figure 11, Mutations in the genes, including *MUC17, TTN, SYNE1, MUC16, FAT4, FLG, CSMD1, FAT3, RYR1, COL6A3, NEB, OBSCN, ZFHX4, TCF7L2, ERN3, NOTCH3, KMT2C,* and *PTRRT,* were detected in both LRRC and PRC samples from five LRRC patients. Mutations in PEG3, NRAS, and BRAF did not occur in the PRC samples of the five patients. In LRRC samples, PEG3 mutations were present in 4 samples, NRAS in 1 sample, and BRAF in 1 sample. The other 27 mutations were present in some of the LRRC or PRC samples.

### The CNVs in PRC and LRRC

After removing the control CNVs from PRC and LRRC samples, the focus was on specific CNVs. As shown in Table 5, *FGFR1* and *MYC* were found to be genes specific for CNVs in PRC and LRRC, respectively. Besides, *FGFR3* CNVS were occurred in both PRC and LRRC. The copy numbers of *FGFR3* in PRC and LRRC tumors were 3.542 and 3.548, respectively. Due to the sample size, after removing control CNVs, no CNVs was found in NRRC. There were no significant CNVs related to Wnt pathway.

**Table 5:** The CNVs of PRC and LRRC samples.

**Sample**	**Chromosome**	**Start**	**End**	**Gene**	**Depth**	**Copy_number**
P1-primary	Chr4	1803536	1803776	FGFR3	677.633	3.12
P2-primary	Chr4	1803536	1803776	FGFR3	493.65	3.04
P3-primary	Chr4	1803536	1803776	FGFR3	699.067	3.27
P3-primary	Chr11	69588723	69588963	FGF4	655.746	3.24
P3-primary	Chr8	38282001	38282241	FGFR1	886.567	3.79
P4-primary	Chr4	1803284	1803510	FGFR3	414.624	4.15
P5-primary	Chr4	1803536	1803776	FGFR3	986.671	4.13
P5-primary	Chr11	69588723	69588963	FGF4	580.725	3.04
P1-recurrent	Chr4	1803284	1803510	FGFR3	273.934	3.71
P2-recurrent	Chr4	1803536	1803776	FGFR3	543.558	3.37
P3-recurrent	Chr4	1803284	1803510	FGFR3	200.765	3.38
P4-recurrent	Chr4	1803536	1803776	FGFR3	772.854	3.92
P4-recurrent	Chr11	69588723	69588963	FGF4	494.054	3.08
P5-recurrent	Chr4	1803536	1803776	FGFR3	472.738	3.36
P5-recurrent	Chr8	128750764	128751028	MYC	2600.53	12.58

## DISCUSSION

WES has a high sensitivity to common, rare, and low-frequency mutations. It could find the most disease-related mutations in the exon region, and only needs to sequence about 1% of the genome [20]. WES had been widely used in CRC to detect the mutational landscape of CRC populations and provided novel insights into the treatment and prognosis of CRC in the clinic [21].

LRRC refers to the recurrence of READ after radical resection. In addition to negative distal and circumferential margins, radical resection also requires no lateral or distant lymph node metastasis. Attention should also be paid to tumor-free operations, including irrigation before anastomosis. However, the evolution of LRRC at the genome level remains unknown. In the present study, WES was performed on PRC, LRRC, and NRRC samples to detect the mutational characteristics of LRRC and discover the specific genes and related pathways. The results of the study showed that LRRC tissues exhibit different mutation profiles.

Many studies have explored the relationship between mutations in key driver genes and CRC metastasis [22]. The specific genes of PRC were found, including *MYCN, SCRIB, SNAPC4,* and *MED15*. *MYCN* is a member of the *MYC* family. The amplification of *MYCN* is related to many tumors, most notably neuroblastoma [23]. A frame shift mutation (c.87dupC) was found in the *MYCN* gene in 5 PRC samples, which was a clinically unknown mutation. *SCRIB* is a membrane protein that is involved in the maintenance of the apical basal cell polarity of epithelial tissues. It plays a tumor-suppressive role in the progression of skin and liver cancer [24]. Notably, a pathogenic mutation of *SCRIB* (c.T233G) was found in both PRC and LRRC samples. *SCRIB* affects tumor development by negatively modulating the Wnt/β-catenin signaling pathway [25]. It is suggested that SCRIB may affect the occurrence and development of READ through the Wnt/β-catenin signaling pathway. *SNAPC4* gene encodes the largest subunit of the small nuclear RNA-activating protein (SNAP) complex, and its role in cancer is unknown. A nonsynonymous mutation was found in the *SNAPC4* gene (c.T3158A) was damaged (SIFT). MED15 is part of the multiprotein mediator complex, which plays a cancer-promoting role in urothelial bladder cancer (BCa) and renal cell carcinoma (RCa).

Two specific genes of LRRC samples were found, including *SCRIB* and *RUSC2*. Non-frameshift mutations of the *RUSC2* (c.831_833del, c.3465_3467del) were found in LRRC samples. *RUSC2* interacts with the SHD domain of GIT2 and reduces GIT2 degradation, which regulates lung cancer progression through EGFR signaling [26]. Whether RUSC2 affects the progression of LRRC through EGFR signaling needs further research.

*FGFR1* and *MYC* were the specific genes of CNVs for PRC and LRRC, respectively. Genetic aberrations in FGFRs have been reported in a variety of cancers, including gastric, lung, and breast cancers [27]. FGFR1 amplification was previously shown to be associated with resistance to endocrine therapy, shorter time to distant metastasis, and shorter overall survival in HR^+^ breast cancer [28]. MYC amplification plays an important role in the progression of CRC [29].

The pathways involved in PRC and LRRC are different. The specific pathways enriched in the PRC were the pentose phosphate pathway, fructose and mannose metabolism, proteoglycans in cancer, ECM-receptor interaction, and protein digestion and absorption. They were involved in the occurrence, development, and metastasis of CRC [30–33]. The pathogenic genes of NRRC were mainly enriched in peroxisome and butanoate metabolism. In CRC tissues, the peroxisome proliferator-activated receptor (PPAR) signaling pathway was down-regulated [34]. Several studies have shown that PPARγ activation promotes cell cycle arrest, apoptosis, and differentiation in many human tumors, and selective synthetic ligands have been shown to act as potential antitumor drugs [35]. It is suggested that the peroxisome limits the LRRC through the PPAR signaling pathway.

In this study, the specific signaling pathways of LRRC were Wnt signaling pathway, gap junction, glucagon signaling pathway, axon guidance, thyroid hormone synthesis, morphine addiction, glycine, serine and threonine metabolism. The present study focused on the Wnt signaling pathway. The *PRICKLE1* mutation (c.C875T) and *WNT6* mutation (c.G629A) were predicted as “D (deleterious)” by SIFT, Polyphen2 and FATHMM software, which were critical to the occurrence of LRRC (Table 4). The expression of LEF1 and cyclin D1 proteins of Wnt signaling pathway was higher in PRC compared to NRRC. Carbonic anhydrase IV (CA4) inhibits the Wnt signaling pathway by targeting the WTAP-WT1-TBL1 axis and is a novel tumor suppressor in CRC [36]. Agrin (AGRN) may act as an oncogenic indicator of READ through activation of the WNT pathway, which could help in the development of optimal treatments for READ [37]. Activation of Wnt signaling as a mechanism of chemoresistance in recurrent small cell lung cancer (SCLC) [38]. PRC1, a novel Wnt target, functions in a positive feedback loop that reinforces Wnt signaling to promote early Hepatocellular carcinoma (HCC) recurrence [39]. AKIP1 is a novel regulator of Wnt/β-catenin signaling and early relapse of HCC [40]. These findings suggest that the development of small molecule drugs that target the Wnt pathway may be important for LRRC.

Neoplastic transformation is frequently associated with a loss of gap junction intercellular communication and a reduction in the expression of connexins in various tumor types [41]. Furthermore, gap junctions may have distinct functional roles in cell growth and cell invasion, as a gap junction inhibitor decreases the invasion of prostate cancer cells [42]. Studies have found that glucagon signaling pathway may be involved in the progression of CRC [43]. Synthesis and growth of tumor proteins can be stimulated by glucagon *in situ* [44]. Several lines of evidence indicate that axon guidance genes are involved not only in neural development but also in cancer development. ROBO1 and ROBO2, crucial regulators of axon guidance, are considered potential tumor suppressor genes [45]. Multiple studies have shown a significant connection between hypothyroidism and pancreatic, gastric, and breast cancer [46]. Deregulation of Thyroid Hormones (THs) system in Colorectal Cancer (CRC) suggests that these hormones may play roles in CRC pathogenesis [47]. Morphine promotes tumorigenesis and cetuximab resistance via EGFR signaling activation in human colorectal cancer [48]. Glycolysis and glycine, serine and threonine were activated in CRC, and these alterations may promote cell proliferation [49].

Besides, this study found two KEGG pathways shared by PRC and LRRC, including signaling pathways regulating pluripotency of stem cells and estrogen signaling pathway. Cancer stem cells (CSCs) are involved in the occurrence and recurrence of CRC and have been identified [50]. Estrogen signaling pathway may be involved in the occurrence of CRC, but the specific mechanism is not yet clear [51]. These two pathways may be involved in the progression from PRC to LRRC.

There are certain limitations in the present study. The number of patients used for this study was too small. Although this study can’t yield a definitive conclusion, Wnt signaling pathway may play a critical role in LRRC. Although we used some tractable methods to validate the possible biological significance of Wnt signaling pathway mutations in LRRC, the specific functions and molecular mechanisms of the Wnt signaling pathway in LRRC need to be further explored.

## CONCLUSIONS

We described the mutation characteristics of LRRC, PRC, and NRRC in the present study. Our results showed that LRRC, PRC, and NRRC had different genomic characteristics and involved different pathways. The signaling pathway of Wnt signaling pathway, Gap junction, Glucagon signaling pathway, Axon guidance, Thyroid hormone synthesis, Morphine addiction, and Serine and threonine metabolism may be related to the occurrence of LRRC. The genes including *NFATC1, PRICKLE1, SOX17,* and *WNT6,* related to Wnt signaling pathway, may play a critical role in LRRC. Further translational and clinical research is imperative to investigate new therapeutic strategies for LRRC.

## Supplementary Material

Supplementary Tables
