# Supplementary material for: Whole-exome sequencing of rectal cancer identifies locally recurrent mutations in the Wnt pathway
Source: Aging (Albany NY). 2021 Oct 12;13(19):23262–83. doi: 10.18632/aging.203618 (PMC8544332; doi:10.18632/aging.203618)
Supplement: Supplementary Tables [file aging-13-203618-s001.pdf]

SUPPLEMENTARY TABLES

Supplementary Table 1. Characteristics of the patients in this study.

Number	Age	Gender	Tumor size, cm	Tumor stage	Pathological description
LRRC (first operation)					
1-LJ	45	male	3	T2N0M0 I	adenocarcinoma
2-LZC	62	male	2	T2N0M0 I	adenocarcinoma
3-SYJ	69	male	2	T4aN0M0 IIB	adenocarcinoma
4-WLJ	57	male	4	T4aN2M0 IIIC	adenocarcinoma
5-WGR	71	male	2	T1N0M0 I	adenocarcinoma
NRRC					
1-SL	49	male	3	T4aN0M0 IIB	adenocarcinoma
2-SCC	61	male	2	T1N0M0 I	adenocarcinoma
3-LHC	70	male	5	T4aN0M0 IIB	adenocarcinoma

Supplementary Table 2. Quality of whole-exome sequencing.

Patient ID	Raw data (Mb)	Q30	[Total] fraction of mapped reads (%)	[Target] average depth
P1-N	17918.2	92.18%	95.58	243.04
P1-P	38812.2	93.14%	92.92	527.11
P1-R	12741.7	93.38%	97.00	187.55
P2-N	17989.6	92.32%	99.23	251.08
P2-P	20155.4	92.78%	98.32	250.19
P2-R	22565.7	92.70%	99.30	319.95
P3-N	18089.1	92.31%	99.10	241.69
P3-P	23193.9	92.85%	98.99	334.37
P3-R	26373.1	92.99%	96.58	370.91
P4-N	17481.1	92.58%	99.20	243.31
P4-P	15413.3	94.65%	96.44	224.44
P4-R	16435.2	95.02%	99.38	232.93
P5-N	16383.6	92.07%	98.67	214.39
P5-P	23762.6	92.84%	98.65	337.59
P5-R	18689.8	93.13%	99.34	247.94
P6-N	26541.1	93.68%	93.56	332.02
P6-NR	29528.5	94.17%	95.81	398.69
P7-N	15592.5	96.32%	93.19	217.23
P7-NR	15828.1	96.18%	93.51	224.44
P8-N	15890.8	95.41%	91.16	181.46
P8-NR	16316.7	96.16%	93.82	196.02

N, Normal rectal cancer; P, Primary rectal cancer; R, Local-recurrent rectal cancer; NR, Non-recurrent rectal cancer.

Supplementary Table 3. Top 15 significant GO terms of BP, MF and CC in PRC samples.

Category	Term	Count	P-Value
BP	positive regulation of transcription from RNA polymerase II promoter	73	9.28E-06
BP	negative regulation of apoptotic process	40	4.49E-05
BP	positive regulation of transcription, DNA-templated	43	7.15E-05
BP	in utero embryonic development	21	1.97E-04
BP	apoptotic cell clearance	6	3.24E-04
CC	integral component of plasma membrane	93	1.35E-05
CC	membrane raft	24	1.82E-05
CC	plasma membrane	217	9.55E-05
CC	endoplasmic reticulum lumen	20	4.54E-04
CC	transcription factor complex	20	4.85E-04
MF	sequence-specific DNA binding	51	1.88E-07
MF	structural molecule activity	28	1.43E-05
MF	ATP binding	98	7.53E-05
MF	steroid hormone receptor activity	10	7.25E-04
MF	protein homodimerization activity	52	8.40E-04

Supplementary Table 4. The KEGG pathways in PRC samples.

Term	Count	%	P-Value
Chronic myeloid leukemia	11	0.009228652	0.003544157
Melanogenesis	13	0.010906589	0.005012796
Signaling pathways regulating pluripotency of stem cells	16	0.013423494	0.005374073
Glioma	10	0.008389684	0.005678579
Platelet activation	15	0.012584526	0.006738809
Acute myeloid leukemia	9	0.007550716	0.00739768
Hepatitis C	15	0.012584526	0.008209968
Oxytocin signaling pathway	16	0.013423494	0.010028696
Estrogen signaling pathway	12	0.010067621	0.012404787
Leukocyte transendothelial migration	13	0.010906589	0.014795069
Pentose phosphate pathway	6	0.00503381	0.015005993
Fructose and mannose metabolism	6	0.00503381	0.022446568
Dilated cardiomyopathy	10	0.008389684	0.027945534
Proteoglycans in cancer	18	0.015101431	0.028185022
Prolactin signaling pathway	9	0.007550716	0.028536839
ECM-receptor interaction	10	0.008389684	0.034080997
ErbB signaling pathway	10	0.008389684	0.034080997
Prostate cancer	10	0.008389684	0.036317726
Protein digestion and absorption	10	0.008389684	0.036317726
HTLV-I infection	21	0.017618336	0.037392779
Rap1 signaling pathway	18	0.015101431	0.042070079
Hypertrophic cardiomyopathy (HCM)	9	0.007550716	0.046279642

Supplementary Table 5. Top 15 significant GO terms of BP, MF and CC in LRRC samples.

Category	Term	Count	P-Value
BP	positive regulation of transcription from RNA polymerase II promoter	65	1.42E-05
BP	retina development in camera-type eye	11	5.71E-05
BP	nervous system development	26	9.90E-05
BP	angiogenesis	22	1.19E-04
BP	negative regulation of transcription from RNA polymerase II promoter	48	2.01E-04
CC	plasma membrane	191	2.91E-05
CC	integral component of plasma membrane	79	6.36E-05
CC	cell-cell junction	17	4.27E-04
CC	mitochondrion	71	5.91E-04
CC	stress fiber	9	5.94E-04
MF	ATP binding	100	3.86E-08
MF	protein tyrosine kinase activity	16	1.75E-04
MF	sequence-specific DNA binding	37	4.22E-04
MF	serine-type endopeptidase activity	21	0.001976808
MF	transmembrane-ephrin receptor activity	4	0.003941594

Supplementary Table 6. Significant pathways in LRRC samples.

Term	Count	%	P-Value
Glucagon signaling pathway	15	0.0149	0.0001
Gap junction	14	0.0139	0.0001
Signaling pathways regulating pluripotency of stem cells	15	0.0149	0.0036
Axon guidance	13	0.0129	0.0106
Metabolic pathways	70	0.0695	0.0163
Neuroactive ligand-receptor interaction	21	0.0209	0.0218
Circadian entrainment	10	0.0099	0.0249
Calcium signaling pathway	15	0.0149	0.0280
Glycine, serine and threonine metabolism	6	0.0060	0.0281
Estrogen signaling pathway	10	0.0099	0.0315
Thyroid hormone synthesis	8	0.0079	0.0351
Wnt signaling pathway	12	0.0119	0.0430
Pathways in cancer	26	0.0258	0.0439
Morphine addiction	9	0.0089	0.0488

Supplementary Table 7. Top 15 significant GO terms of BP, MF, and CC in NRRC samples.

Category	Term	Count	P-Value
BP	GO:0006810~transport	12	0.001550891
BP	GO:0001937~negative regulation of endothelial cell proliferation	4	0.003777034
BP	GO:0042127~regulation of cell proliferation	8	0.004251828
BP	GO:0010628~positive regulation of gene expression	9	0.008125803
BP	GO:2001241~positive regulation of extrinsic apoptotic signaling pathway in absence of ligand	3	0.009862223
CC	GO:0005777~peroxisome	6	0.004372107
CC	GO:0005759~mitochondrial matrix	10	0.006789772
CC	GO:0005887~integral component of plasma membrane	25	0.010268471
CC	GO:0005576~extracellular region	27	0.013785056
CC	GO:0016020~membrane	34	0.015904909
MF	GO:0005179~hormone activity	6	0.003657841
MF	GO:0050662~coenzyme binding	3	0.007258267
MF	GO:0001105~RNA polymerase II transcription coactivator activity	4	0.007540464
MF	GO:0005524~ATP binding	27	0.011626528
MF	GO:0016887~ATPase activity	7	0.015231878

Supplementary Table 8. Significant pathways in NRRC samples.

Term	Count	%	P-Value
Peroxisome	5	0.017031133	0.021363124
Metabolic pathways	24	0.081749438	0.030427677
Butanoate metabolism	3	0.01021868	0.046182301
